# Recruitment strategies and geographic representativeness for patient survey studies in rare diseases: Experience from the living with myeloproliferative neoplasms patient survey

**DOI:** 10.1371/journal.pone.0243562

**Published:** 2020-12-31

**Authors:** Jingbo Yu, Dilan Paranagama, Shreekant Parasuraman

**Affiliations:** Incyte Corporation, Wilmington, DE, United States of America; Cleveland Clinic, UNITED STATES

## Abstract

**Background:**

Recruitment of individuals with rare diseases for studies of real-world patient-reported outcomes is limited by small base populations. Myeloproliferative neoplasms (MPNs) are a group of rare, chronic, hematologic malignancies. In this study, recruitment strategies and geographic representativeness from the Living with MPNs survey are reported.

**Methods:**

The Living with MPNs online cross-sectional survey was conducted between April and November 2016. Individuals 18 to 70 years of age living in the United States and diagnosed with an MPN were eligible to participate. Recruitment approaches included direct contact via emails and postcards; posts on MPN-focused social media and patient advocacy websites; postcard mailings to doctors’ offices; and advertisements on medical websites, Google, and Facebook. Geographic representativeness was assessed based on the number of survey respondents living in each state or the District of Columbia and by the number of survey respondents per 10 million residents.

**Results:**

A total of 904 respondents with MPNs completed the survey. The recruitment method yielding the greatest number of respondents was advertisements on MPN-focused social media (47.6% of respondents), followed by emails (35.1%) and postcards (13.9%) sent through MPN advocacy groups. Home state information was provided by 775 respondents from 46 states (range of respondents per state, 1–89). The number of respondents per 10 million residents in the 46 states with respondents ranged from 12.1 to 52.7.

**Conclusions:**

Recruitment using social media and communications through patient groups and advocacy organizations are effective in obtaining geographically representative samples of individuals with MPNs in the United States. These approaches may also be effective in other rare diseases.

## Introduction

Rare diseases, defined in the United States as conditions affecting fewer than 200,000 people at any given time [1], collectively represent a substantial societal burden and can significantly reduce quality of life for patients and caregivers. Patient-reported outcomes are critical to understanding the burden faced by individuals with rare diseases; however, small populations and lack of clinical knowledge and specialized care centers make it difficult to collect widely representative data [1]. Although recruitment strategies for surveys of individuals with highly prevalent diseases may include prospective recruitment at participating institutions [2–4] or identification of patients in existing medical claims databases [5], traditional methods are often inadequate for rare diseases. As such, communications from patient advocacy groups and postings or advertisements on social media websites may become valuable avenues for recruiting individuals with rare diseases [6, 7].

Myeloproliferative neoplasms (MPNs) are a group of rare, chronic, hematologic malignancies that include myelofibrosis (MF), polycythemia vera (PV), and essential thrombocythemia (ET). The age-adjusted prevalence rates for MF, PV, and ET in the United States are 4 to 6, 45 to 57, and 39 to 57 per 100,000, respectively [8]. Overall survival is reduced among individuals with MPNs compared with the general population [9, 10]. Individuals with MPNs face a pronounced symptom burden that typically includes fatigue, night sweats, bone pain, itching, problems with concentration, and splenomegaly-related symptoms [11, 12]. Furthermore, MPNs can negatively affect work ability and productivity, as well as overall quality of life [13, 14].

The Living with MPNs survey was designed to evaluate the impact of MPNs on employment and everyday life in the United States [13]. A variety of recruitment strategies were used to target a widely representative population. The objective of this analysis was to describe recruitment strategies and assess geographic representativeness of respondents in the Living with MPNs survey. The approach described here will be applicable to other rare diseases and may help to inform patient-centered care.

## Materials and methods

### Study design and participants

The Living with MPNs survey was a cross-sectional web-based questionnaire that has been previously described [13, 15]. In brief, individuals 18 to 70 years of age with a diagnosis of MF, PV, or ET and living in the United States were eligible to participate. The online survey was conducted between April and November 2016, and participants were offered an optional $25 incentive for completing the survey. Survey recruitment strategies consisted of posts on MPN-focused social media and patient advocacy websites; text or banner advertisements on selected medical websites, search engines (Google), and social media sites (Facebook); emails or postcards sent through MPN advocacy groups; and postcards sent to the offices of hematologists and oncologists for distribution. The survey was designed to take approximately 30 minutes to complete and included 100 questions evaluating patient demographics; MPN diagnosis and disease-related medical history; and the impact of MPNs on employment, work productivity, and activities of daily living. The Living with MPNs survey was approved by the Quorum Review Institutional Review Board, and all respondents provided written informed consent electronically (by selecting “agree” on the consent page) before starting the survey. A copy of the full survey is available in S1 Appendix.

### Assessments and statistical analyses

Geographic representativeness was assessed based on the number of survey respondents living in each state or the District of Columbia and by the number of survey respondents per 10 million residents of each state or the District of Columbia, based on the 2015 population according to US Census Bureau National and State Population Estimates. Data were described using summary statistics. No formal statistical tests were conducted in the analysis.

## Results

### Patient recruitment and demographics

**Fig 1:**
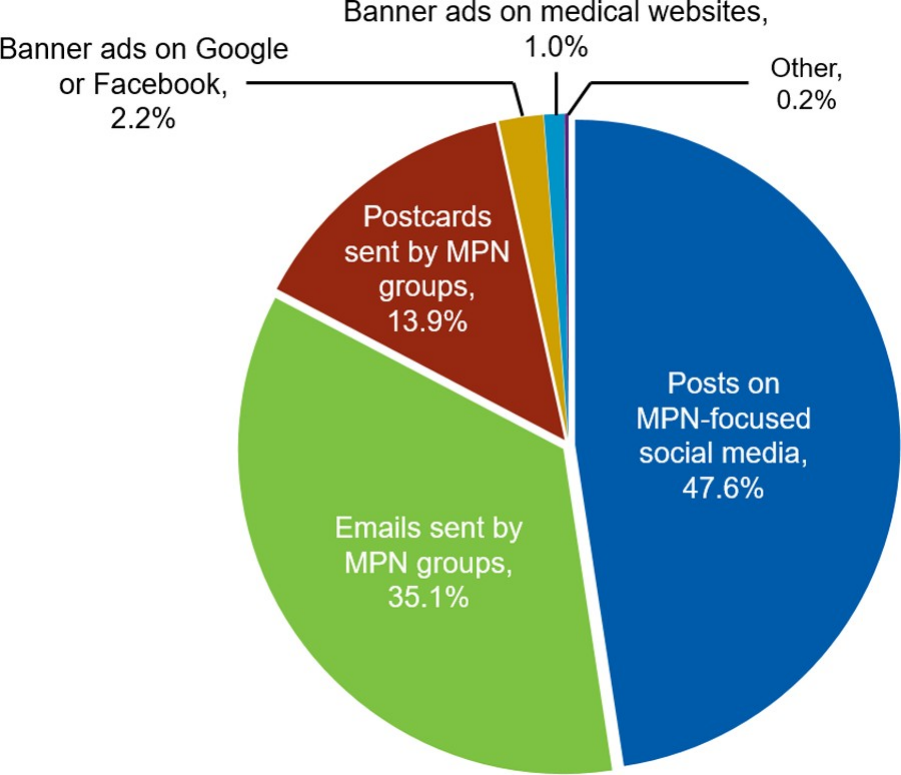
Recruitment method. MPN, myeloproliferative neoplasm.

A total of 904 respondents with MPNs completed the survey, with 779 (86.2%) accepting the optional $25 incentive. The recruitment approach yielding the greatest number of respondents was survey advertisements in MPN-focused social media groups (47.6% of respondents), followed by emails and postcards sent to patients by MPN groups (35.1% and 13.9% of respondents, respectively; Fig 1). In total, 96.6% of respondents were recruited through MPN patient groups, whereas banner advertisements on medical websites, Google, or Facebook methods accounted for just 3.2% of overall recruitment.

Two-hundred seventy respondents (29.9%) reported a diagnosis of MF, 393 (43.5%) reported a diagnosis of PV, and 241 (26.7%) reported a diagnosis of ET. The mean (SD) reported age of all respondents was 55.1 (10.9) years; most respondents (70.9%) were 40 to 65 years of age at the time of the survey. Most respondents (73.2%) were female, 46.1% reported ≥1 comorbid condition, and 65.5% were employed full- or part-time at the time of MPN diagnosis. At the time of the survey, the mean (SD) reported MPN disease duration for all respondents was 5.7 (6.4) years.

### Geographic distribution of respondents

**Fig 2:**
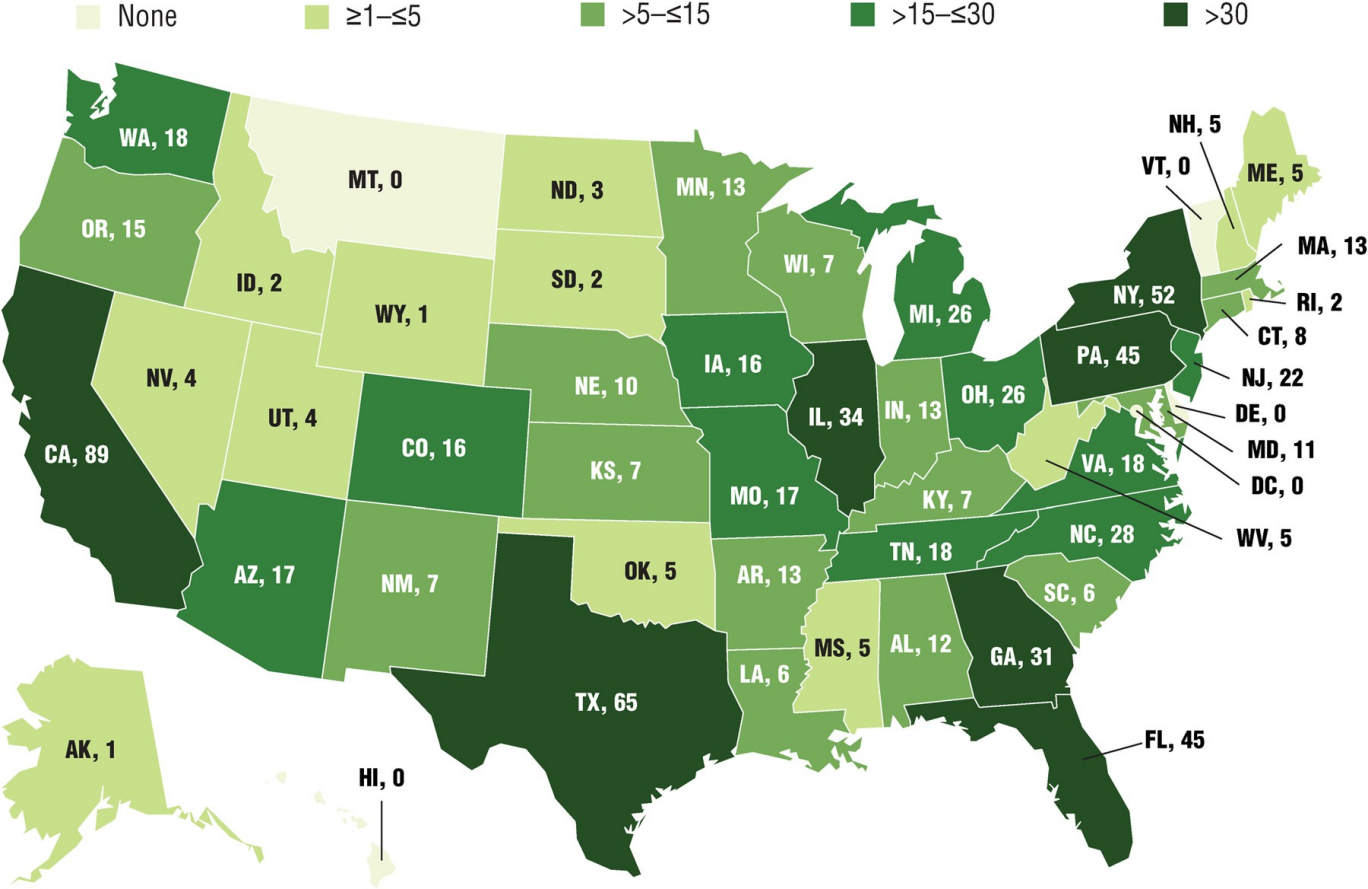
Number of survey respondents in each state. 775 respondents provided home state information.

Home state information was provided by 775 respondents, representing 46 states; the number of respondents from each of the 46 states ranged from 1 to 89. The states with the most respondents were California (n = 89), Texas (n = 65), New York (n = 52), Florida (n = 45), and Pennsylvania (n = 45; Fig 2). Seven states had >30 respondents, 11 states had >15 to ≤30 respondents, 15 states had >5 to ≤15 respondents, and 13 states had ≥1 to ≤5 respondents. No respondents reported being from Delaware, Hawaii, Montana, Vermont, or the District of Columbia.

**Fig 3:**
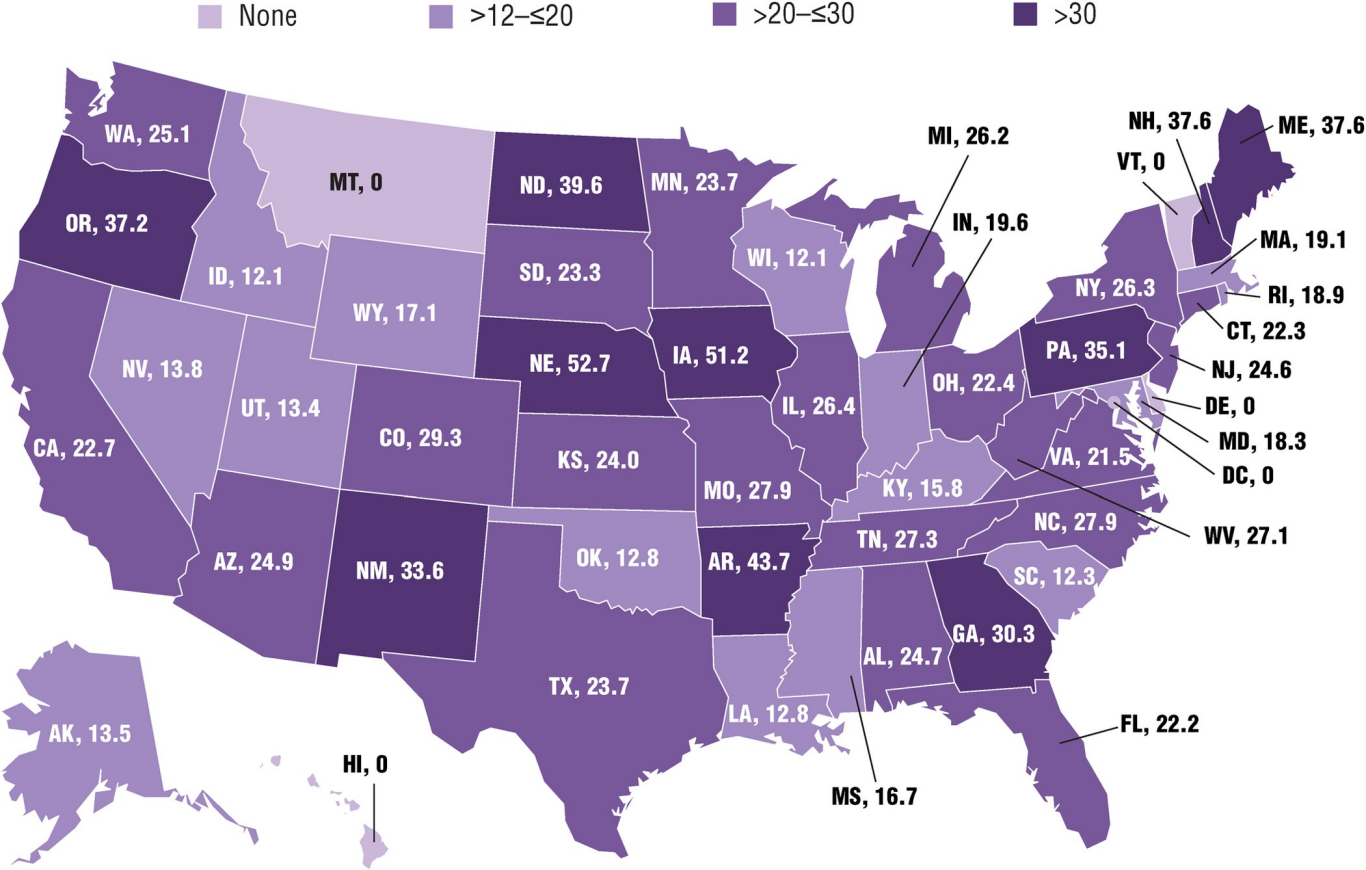
Survey respondents per 10 million residents in each state. 775 respondents provided home state information.

The number of respondents per 10 million residents in the 46 states with survey participants ranged from 12.1 to 52.7 (Fig 3). The states with the highest response rates per 10 million residents were Nebraska (52.7), Iowa (51.2), Arkansas (43.7), North Dakota (39.6), Maine (37.6), and New Hampshire (37.6). Ten states had >30 individuals per 10 million residents who responded to the survey, 21 states had >20 to ≤30 respondents per 10 million residents, and 15 states had >12 to ≤20 respondents per 10 million residents.

## Discussion

The Living with MPNs survey attracted more than 900 respondents with MPNs from 46 states. The vast majority of respondents (95%) were recruited through MPN patient groups, and about half of these participants were recruited via social media posts. In fact, MPN-focused social media was the most effective recruitment method for this rare disease survey. These findings are supported by other studies demonstrating that social media recruitment, particularly via Facebook and when in partnership with advocacy organizations, increases accessibility to target populations and yields higher response rates in understudied and demographically-diverse populations [16–18]. In a previous study of recruitment strategies for pediatric patients with Klinefelter syndrome, web-based and social networking strategies successfully recruited a greater proportion of participants (91%) than traditional approaches (letters, brochures, clinic referral; 9%) [19]. Geographic distance from the study site was one of the most frequent reasons for declining participation (22%) [19]. Accordingly, web-based recruitment methods and social media posts, such as those employed in the Living with MPNs survey, may offer several advantages compared with traditional methods (eg, face-to-face interviews with treating physicians or recruitment of patients through registry databases). Web-based approaches may increase survey participation, which is crucial for understanding rare disease states, by allowing patient self-identification, increased accessibility, and reduced time burden [20], as well as increased willingness to participate due to anonymity of the online platform [21]. In agreement with these interpretations, a previous study of symptom burden in individuals with ankylosing spondylitis demonstrated that review of social media posts captured a greater number of symptoms than surveys conducted during face-to-face interviews or through group concept mapping [21].

The use of social media to recruit respondents is associated with limitations, however, including inability to validate diagnosis and demographic information [20]. Recruitment by advocacy groups (eg, targeted emails) may avoid self-identification bias because individuals belonging to such groups are often referred by treating physicians after diagnosis. In the Living with MPNs survey, the proportions of patients diagnosed with MF (30%), PV (44%), and ET (27%) were comparable to those from the US patient population of the MPN Landmark survey (MF, 25%; PV, 47%; ET, 28%) [11]. The MPN Landmark survey had a similar geographic distribution as the Living with MPNs survey, including respondents from 47 states and the District of Columbia, although detailed geographic information was not reported.

Limitations of this study included potential selection bias regarding who completed the study. Furthermore, participant recruitment was limited to individuals who could be reached via email, postcard mailings, social media, and Google search advertising. Geographic data collected did not include necessary information to stratify patients by urban and rural regions. Finally, demographic data were not compared among recruitment strategies to assess which strategy resulted in the most representative patient sample.

## Conclusions

Analysis of recruitment data from the Living with MPNs survey suggests that recruitment strategies using patient groups, advocacy organizations, and social media are effective for obtaining geographically representative samples of individuals with MPNs in the United States. These findings may extend to other rare or orphan disease states in which traditional patient recruitment methods are limited.

## Supporting information

S1 Appendix Living with MPNs patient survey. (PDF)
