# Supplementary material for: Recruitment strategies and geographic representativeness for patient survey studies in rare diseases: Experience from the living with myeloproliferative neoplasms patient survey
Source: PLoS One. 2020 Dec 31;15(12):e0243562. doi: 10.1371/journal.pone.0243562 (PMC7774910; doi:10.1371/journal.pone.0243562)
Supplement: S1 Appendix — (PDF) [file pone.0243562.s001.pdf]

Living with MPNs Survey

Full Questionnaire

May 20, 2016

STUDY INFORMATION AND ENTRY PAGE – LIVING WITH MPNS SURVEY

Welcome

Thank you for your interest in participating in this health survey for myeloproliferative neoplasm (MPN) diseases which include myelofibrosis (MF), polycythemia vera (PV), essential thrombocythemia (ET).

The purpose of this study is to learn more about your experiences living with an MPN, its impact on your general health concerns, quality of life, work productivity, and the related financial impact to you and your family.

- This survey includes questions on disease diagnosis, signs and symptoms, impact on everyday life and work productivity, treatments, and demographic information
- In order to estimate the financial impact of the disease, a few questions will be asked about your annual income and out of pocket costs related to the disease

ALL OF THESE DATA WILL BE KEPT CONFIDENTIAL

The survey should take approximately 30 minutes to complete. This survey is being conducted by HRA on behalf of Incyte Corporation who is the sponsor of the study. Quorum Review (an institutional review board) has reviewed and approved the survey.

This study should not involve any physical risk to you. There is no study medication involved, and you will not receive any medical benefits from participating in this study. Nothing will change about your regular medical care whether or not you participate in this study.

Your participation is voluntary. If you decide to take this survey, please try to answer ALL the questions, if you do not feel comfortable answering a question, you can use the exit button to close the survey at any time (located to the right of the title banner that says "Living with MPNs Survey").

Your privacy is protected. HRA will not share your personally identifiable information with anyone without your permission. Your responses to this survey are also confidential. Publications resulting from this research will not individually identify you. Please see HRA's Privacy Policy for additional information www.hrainc.net.

If you are eligible and complete the entire survey you may choose to receive a $25 gift card. The first few questions will determine if you are eligible to complete the full survey. At the end of the survey you will be asked to provide your mailing address. This information will ONLY be used for this study and for the purposes of mailing the $25 gift card and will NOT be used for any other purpose. Please note that if you leave the survey early and not complete all the questions, you will not receive any payment/gift card.

You can only take this survey once.

If you have any questions about this survey or issues using the survey, please call HRA toll free at (855) 563-6341 for assistance. To save a full printed copy of this study information, please visit www.hrainc.net to view or save a copy of the Participant Information Sheet.

By clicking “Accept”, you understand and agree to the following:

- I have read and understood this information about this health survey provided here
- I am at least 18 years old and under the age of 70
- I live in the United States
- I understand the use of the information I provide in the survey

◯ Accept
◯ Decline

Instructions

Please read the following instructions before beginning this survey.

The survey will take about 30 minutes to complete. When taking the survey, try to identify a location that will allow you to concentrate on your responses and complete the entire survey in one sitting on a single device. If you need to take a break, please leave the internet browser window open so you can return to the same point in the survey.

The survey is best accessed via a desktop, laptop, or tablet. While discouraged, if you are taking the survey on a handheld mobile device you may need to hold the device horizontally for a better view. Please follow the prompts in the survey.

TO MOVE FORWARD:

Click on the NEXT button located at the bottom of the page to save your responses and continue to the next page.

TO MOVE BACK:

Use the PREV button located at the bottom of the page to view your responses on a previous page. You may change your answers to previously entered responses. Do not use the BACK button of your browser to return to the previous page; this may cause you to exit the survey, and lose your responses.

TO EXIT:

If you are deemed ineligible to take the survey, you will see a closing screen, at which point you may close your web browser. If you are eligible and you complete the survey, you will see a closing screen, at which point you may close your web browser. You may also exit at any time by using the Exit button located to the right of the title banner that says "Living with MPNs Survey".

Screener Questions

DIAGNOSIS SCREEN

ASK ALL

S1. How old are you? **[REQUIRED]**

____________ //RANGE=10-100// [100=100 or more]

ASK IF S1<18 OR S1>70

TERM1. Thank you for your time. Unfortunately, you do not qualify to take the survey. You may close this browser window at any time.

ASK IF S1 >18

S2a. What is your current diagnosis? (Please pick one) **[REQUIRED]**

01 myelofibrosis
02 polycythemia vera
03 essential thrombocythemia
04 none of these
77 don't know

ASK IF S2A=04 OR 77

TERM2. Thank you so much for your time. Unfortunately, you do not qualify to take the survey. You may close this browser window at any time.

ASK IF S2A=01 [REQUIRED]

S2b. How old were you when your doctor first diagnosed your **myelofibrosis**?

_______ //RANGE=0-70//

ASK IF S2A=01 OR 02

S3a. Has a doctor ever diagnosed you with **polycythemia vera**?

IMPORTANT NOTE: **polycythemia vera** (PV) is a malignant blood disorder in which the bone marrow makes too many red blood cells, and is part of a set of diseases known as myeloproliferative neoplasm (MPN). PV is **different** from other types of *polycythemia*, sometimes called *secondary polycythemia*, which may ba caused by smoking, living or visiting high elevation places, COPD, and testosterone use. Secondary polycythemia can also result in too many red blood cells, but is distinct from polycythemia vera.

01 Yes
02 No
77 Don't know

ASK IF S3A=01 OR S2A=02 [REQUIRED]

S3b. How old were you when your doctor first diagnosed your **polycythemia vera**?

_______ //RANGE=0-70//

ASK IF S2A=01

S4a. Has a doctor ever diagnosed you with **essential thrombocythemia**?

01 Yes
02 No
77 Don't know

ASK IF S4A=01

S4b. How old were you when your doctor first diagnosed your **essential thrombocythemia**?

_______ //RANGE=1-100//

ASK ALL

S4. Do you live in the United States? [REQUIRED]

01 Yes
02 No

ASK IF S4=02

TERM3. Thank you so much for your time. Unfortunately, you do not qualify to take the survey. You may close this browser window at any time.

Questions

ASK ALL

Q1. What is your gender?

01 Male
02 Female

Q2. What is the last year or grade of school you completed?

01 Less than high school diploma
02 High school graduate, no college
03 Some college or associate degree
04 Bachelor's degree
05 Advanced degree

Q3. Did the doctor treating you for [**S2A**] classify your current condition with a particular risk score? (For example: high, intermediate, low) **[REQUIRED]**

01 No
02 Yes – High risk
03 Yes – Intermediate risk
04 Yes – Low risk
05 Yes – Do not recall risk level

Q4. Have you ever been diagnosed with any of the following? (*Mark all that apply*) **[REQUIRED]**

01 Myocardial infarction
02 Congestive heart failure
03 Ischemic stroke
04 Transient ischemic attack
05 Deep vein thrombosis
06 Pulmonary embolism
08 Other blood cancer (for example, leukemia, malignant lymphoma, myeloma, etc)
09 Solid tumor (for example, breast cancer, lung cancer, prostate cancer, or other non blood cancer)
10 Metastatic solid tumor
11 Diabetes
12 Emphysema/ chronic obstructive pulmonary disease(COPD)/chronic bronchitis
13 Connective tissue disorders (lupus, rheumatoid arthritis, psoriasis, etc.)
14 Moderate to severe kidney disease
15 Liver disease
16 Hemiplegia
17 Dementia
18 Peptic ulcer disease
19 AIDS (i.e., Acquired Immune Deficiency Syndrome)
20 Other
21 None

ASK ALL

Q5. What was your employment situation when you were diagnosed with [**S2A**]? **[REQUIRED]**

01 Employed full-time (40 hours a week)
02 Employed part-time (less than 40 hours a week)
03 Unemployed seeking employment
04 Unemployed not seeking employment
05 Retired
06 Self-employed
07 Disabled
08 Home maker
09 Other

ASK IF Q5=01, 02, 03, 04, 05, 06, 07, 09

Q6. How much was your annual income (before tax; please include wage/salary and other income; not including income by other family members)when you were diagnosed with [**S2A**]? **[REQUIRED] [FORMATTED AS A DROP DOWN MENU]**

01 Less than $9,999
02 $10,000 to $19,999
03 $20,000 to $29,999
04 $30,000 to $39,999
05 $40,000 to $49,999
06 $50,000 to $59,999
07 $60,000 to $69,999
08 $70,000 to $79,999
09 $80,000 to $89,999
10 $90,000 to $99,999
11 $100,000 to $109,999
12 $110,000 to $119,999
13 $120,000 to $129,999
14 $130,000 to $139,999
15 $140,000 to $149,999
16 $150,000 to $159,999
17 $160,000 to $169,999
18 $170,000 to $179,999
19 $180,000 to $189,999
20 $190,000 to $199,999
21 $200,000 to $224,999
22 $225,000 to $249,999
23 $250,000 to $274,999
24 $275,000 to $299,999
25 $300,000 and above

ASK IF Q5= 1 OR 2 [REQUIRED]

Q7. As a result of your [**S2A**], have you ever left a job?

01 Yes

02 No

ASK IF Q7=01

Q8. How many times have you left a job as a result of your [**S2A**]?

01 1 time

02 2 times

03 More than 2 times

ASK IF Q8=01

Q9a.

Please enter the year that you left that job as a result of your [**S2A**].	//RANGE=1945-2016//

ASK IF Q8=02

Q9b.

Please enter the year for the 1st time you left a job as a result of your [**S2A**].	//RANGE=1945-2016//
Please enter the year for the 2nd time you left a job as a result of your [**S2A**].	//RANGE=1945-2016//

ASK IF Q8=03

Q9c. Please enter the years for the times you left a job as a result of your [**S2A**].

What was the year for the 1st time?	//RANGE=1945-2016//
What was the year for the 2nd time?	//RANGE=1945-2016//
What was the year for the most recent time?	//RANGE=1945-2016//

The following questions ask about **the most recent time** when you left a job due to [**S2A**]

ASK IF Q7=01

Q10. About **the most recent time when you left a job** due to [**S2A**]:

Was the job you left most recently a full-time or part-time job?

01 Full-time

02 Part-time

ASK IF Q7=01

Q11. About **the most recent time when you left a job** due to [**S2A**]:

How much was your annual salary/wage at the time you left?

[same format for other income/salary questions]

ASK IF Q7=01

Q11a1. About **the most recent time when you left a job** due to [**S2A**]:

Did you experience a **change in health insurance coverage** due to leaving this job?

01 Yes

02 No

ASK IF Q11A1=01

Q11a2. What was the **change in health insurance coverage**?

01 Switched from employer sponsored plan to COBRA

02 Switched from employer sponsored plan to Medicare or Medicaid

03 Switched from employer sponsored plan to self-purchased insurance

04 Switched to a new employer sponsored insurance plan

05 Became uninsured

06 Other

ASK IF Q11A1=01

Q11a3. Compared to your previous health insurance coverage, did your **new insurance costs change**?

01 Paid less for my yearly premium and out of pocket costs (e.g., copay, deductible)

02 No change in insurance premium and out of pocket costs

03 My yearly premium and/or yearly out of pocket costs increased by $1 to $1000

04 My yearly premium and/or yearly out of pocket costs increased by $1000 to $5,000

05 My yearly premium and/or yearly out of pocket costs increased by more than $5,000

06 Do not recall

ASK IF Q11A1=01

Q11a4. Since you left the job, have you made any **significant changes in your living arrangement or life style**? (*Mark all that apply*)

01 Made modification in living arrangements (e.g., sold house, downsized house, or rental)

02 Cut back on everyday living costs (e.g., clothes, food, car)

03 Filed for bankruptcy

04 Other (Please specify)

ASK IF Q7=01

Q12. About **the most recent time when you left a job** due to [**S2A**]:

After you left, did you find another permanent job?

01 No, I did not look for another permanent job [GO TO Q15]

02 No, I am still looking for another permanent job [GO TO Q22]

03 Yes, I took a full-time position

04 Yes, I took a part-time position

ASK IF Q12=03 OR 04

Q13. About **the most recent time when you left a job** due to [S2A]:
After you left, how long did it take you to find this new position?

Years (NOT REQUIRED) allow blanks without validation)	Months (NOT REQUIRED – allow blanks without validation)
//RANGE=1-70//	//RANGE=0-11//

ASK IF Q12=03 OR 04

Q14. How much was your annual salary/wage at this new job?
[same format for other income/salary questions]

ASK IF Q12=03 OR 04

Q15. How many years in advance did you leave the job compared to the retirement age you had originally planned?

_________ years //RANGE=0-65//

ASK IF Q7= 02

Q16. Have you ever seriously considered leaving your job because of your [S2A]?

01 Yes
02 No

ASK IF Q16=01

Q17. What was your reason for staying in that job? (*Mark all that apply*)

01 Needed health insurance
02 For continuity of benefits (other than health insurance)
03 Primary source of income for household
04 Needed additional income
05 Other

ASK IF Q7=02

Q18. Have you ever seriously considered taking medical disability leave because of your [S2A]?

01 Yes
02 No

ASK IF Q18=01

Q19. What was your reason for not taking medical disability leave? (*Mark all that apply*)

01 Needed health insurance
02 For continuity of benefits (other than health insurance)
03 Primary source of income for household
04 Needed additional income
05 Other

Ask if Q7=02

Q20. Have you ever seriously considered changing from full-time to part-time employment, or reducing work hours, or transferring to a less demanding job because of your [**S2a**]?

01 Yes
02 No

Ask if Q20=01

Q21. What was your reason for not making the considered employment change? (*Mark all that apply*)

01 Needed health insurance
02 For continuity of benefits (other than health insurance)
03 Primary source of income for household
04 Needed additional income
05 There were no other job opportunites that I was qualified for
06 Other

ASK IF Q5= 01 OR 02

Q22. As a result of your [S2A], have you ever taken early retirement?

01 Yes

02 No

ASK IF Q22=01

Q23. When did you take early retirement?

Month	Year (4 digit) (REQUIRED) Validation: Please enter both a month and year
//RANGE=January through December//	//RANGE=1960-2016//

ASK IF Q22=01

Q24. Did you retire from a full-time or part-time job?

01 Full-time

02 Part-time

ASK IF Q22=01

Q25. How much was your annual salary/wage at the time you left?

[same format for other income/salary questions]

ASK IF Q22=01

Q26. How many years in advance did you retire compared to what you had originally planned?

_________ years //RANGE=0-65//

ASK IF Q22=01

Q26a1. Did you experience a **change in health insurance coverage** when you took early retirement?

01 Yes

02 No

ASK IF Q26A1=01

Q26a2. What was the **change in health insurance coverage**?

01 Switched from employer sponsored plan to COBRA

02 Switched from employer sponsored plan to Medicare or Medicaid

03 Switched from employer sponsored plan to self-purchased insurance

04 Switched to a new employer sponsored insurance plan

05 Became uninsured

06 Other

ASK IF Q26A1=01

Q26a3. Compared to your previous health insurance coverage, did your **new insurance costs change**?

01 Paid less for my yearly premium and out of pocket costs (e.g., copay, deductible)
02 No change in insurance premium and out of pocket costs
03 My yearly premium and/or yearly out of pocket costs increased by $1 to $1000
04 My yearly premium and/or yearly out of pocket costs increased by $1000 to $5,000
05 My yearly premium and/or yearly out of pocket costs increased by more than $5,000
06 Do not recall

ASK IF Q26A1=01

Q26a4. Since you took early retirement, have you made any **significant changes in your living arrangement or life style**? (*Mark all that apply*)

01 Made modification in living arrangements (e.g., sold house, downsized house, or rental)
02 Cut back on everyday living costs (e.g., clothes, food, car)
03 Filed for bankruptcy
04 Other (Please specify)

ASK IF Q16= 02

Q27. Have you ever seriously considered taking early retirement because of your [**S2A**]?

01 Yes
02 No

ASK IF Q27=01

Q28. What was your reason for staying in that job? (Select all that applies)

01 Needed health insurance
02 For continuity of benefits (other than health insurance)
03 Primary source of income for household
04 Needed additional income
05 Other

ASK IF Q5= 01 OR 02

Q29. As a result of your [S2A], have you ever gone on medical disability leave (including short term disability, long term disability, or other disability)?

01 Yes

02 No

ASK IF Q29=01

Q30. How many times have you ever gone on **medical disability leave** (including short term disability, long term disability, or other disability) as a result of your [S2A]?

01 1 time

02 2 times

03 More than 2 times

ASK IF Q30=01

Q31a.

Please enter the year that you went on **medical disability leave** as a result of your [S2A].	//RANGE=1945-2016//

ASK IF Q30=02

Q31b.

Please enter the year for the 1st time you went on **medical disability leave** as a result of your [S2A].	//RANGE=1945-2016//
Please enter the year for the 2nd time you went on **medical disability leave** as a result of your [S2A].	//RANGE=1945-2016//

ASK IF Q30=03

Q31c. Please enter the years for the times you went on **medical disability leave** as a result of your [S2A].

What was the year for the 1st time?	//RANGE=1945-2016//
What was the year for the 2nd time?	//RANGE=1945-2016//
What was the year for the most recent time?	//RANGE=1945-2016//

The following questions ask about **the most recent time you went on medical disability due to your [**S2A**].**

ASK IF Q29=01

Q32. Was that a full-time or part-time job?

01 Full-time

02 Part-time

ASK IF Q29=01

Q33. About **the most recent time you went on medical disability** due to your [S2A]:

Did you take the leave as short-term or long-term disability leave?

01 Short-term disability leave

02 Long-term disability leave

ASK IF Q29=01

Q34. About **the most recent time you went on medical disability** due to your [**S2A**]:
How much was your annual salary/wage from your employer at the time you left on disability?
[same format for other income/salary questions]

ASK IF Q29=01

Q34a1. Did you experience a **change in health insurance coverage** when you went on medical disability leave?

01 Yes
02 No

ASK IF Q34A1=01

Q34a2. What was the **change in health insurance coverage**?

01 Switched from employer sponsored plan to COBRA
02 Switched from employer sponsored plan to Medicare or Medicaid
03 Switched from employer sponsored plan to self-purchased insurance
04 Switched to a new employer sponsored insurance plan
05 Became uninsured
06 Other

ASK IF Q34A1=01

Q34a3. Compared to your previous health insurance coverage, did your **new insurance costs change**?

01 Paid less for my yearly premium and out of pocket costs (e.g., copay, deductible)
02 No change in insurance premium and out of pocket costs
03 My yearly premium and/or yearly out of pocket costs increased by $1 to $1000
04 My yearly premium and/or yearly out of pocket costs increased by $1000 to $5,000
05 My yearly premium and/or yearly out of pocket costs increased by more than $5,000
06 Do not recall

ASK IF Q34A1=01

Q34a4. Since you went on medical disability leave, have you made any **significant changes in your living arrangement or life style**? (*Mark all that apply*)

01 Made modification in living arrangements (e.g., sold house, downsized house, or rental)
02 Cut back on everyday living costs (e.g., clothes, food, car)
03 Filed for bankruptcy
04 Other (Please specify)

ASK IF Q29=01

Q35. About **the most recent time you went on medical disability** due to your [**S2A**]:
After you left on disability, did you return to that job?

01 No, I did not return [GO TO Q30]
02 Yes, I returned to a full-time position
03 Yes, I returned to a part-time position

ASK IF Q35=02 OR 03

Q36. About **the most recent time you went on medical disability** due to your [**S2A**]:

How long did it take you to return to that job?

Years (NOT REQUIRED) allow blanks without validation)	Months (NOT REQUIRED – allow blanks without validation)
//RANGE=1-70//	//RANGE=0-11//

ASK IF Q35=02 OR 03

Q37. About **the most recent time you went on medical disability** due to your [**S2A**]:
How much was your annual salary/wage from your employer when you returned to that job?
[same format for other income/salary questions]

ASK ALL

Q38. Have you ever applied for Social Security disability benefit due to your [**S2A**]?

01 Yes

02 No

ASK IF Q38=01

Q39. Have you ever received Social Security disability benefit due to your [**S2A**]?

01 Yes

02 No

ASK IF Q5= 01 OR 02

Q40. As a result of your [S2A], have you ever **changed from full-time employment to part-time employment**?

01 Yes
02 No

ASK IF Q40=01

Q41. How many times have you ever **changed from full-time to part-time employment** as a result of your [S2A]?

01 1 time
02 2 times
03 More than 2 times

ASK IF Q41=01

Q42a.

Please enter the year that you **changed from full-time to part-time employment** as a result of your [S2A].	//RANGE=1945-2016//

ASK IF Q41=02

Q42b.

Please enter the year for the 1st time you **changed from full-time to part-time employment** as a result of your [S2A].	//RANGE=1945-2016//
Please enter the year for the 2nd time you **changed from full-time to part-time employment** as a result of your [S2A].	//RANGE=1945-2016//

ASK IF Q41=03

Q42c. Please enter the years for the times you **changed from full-time to part-time employment** as a result of your {{ Q3 }}.

What was the year for the 1st time?	//RANGE=1945-2016//
What was the year for the 2nd time?	//RANGE=1945-2016//
What was the year for the most recent time?	//RANGE=1945-2016//

The following questions ask about **the most recent time you changed from full-time to part-time employment** due to your **[**S2A**].**

ASK IF Q40=01

Q43. How much was your annual salary/wage at the time you changed from full-time to part-time?

[same format for other income/salary questions]

ASK IF Q40=01

Q44. About the **most recent time you changed from full-time to part-time employment** due to your **[**S2A**]:**

How many hours per week (on average) were you working before you changed to part-time?

__[RANGE=1-60]__________ ~~per week~~

ASK IF Q40=01

Q44a1. Did you experience a **change in health insurance coverage** when you changed from full-time to part-time employment?

01 Yes
02 No

ASK IF Q44A1=01

Q44a2. What was the **change in health insurance coverage**?

01 Switched from employer sponsored plan to COBRA
02 Switched from employer sponsored plan to Medicare or Medicaid
03 Switched from employer sponsored plan to self-purchased insurance
04 Switched to a new employer sponsored insurance plan
05 Became uninsured
06 Other

ASK IF Q44A1=01

Q44a3. Compared to your previous health insurance coverage, did your **new insurance costs change**?

01 Paid less for my yearly premium and out of pocket costs (e.g., copay, deductible)
02 No change in insurance premium and out of pocket costs
03 My yearly premium and/or yearly out of pocket costs increased by $1 to $1000
04 My yearly premium and/or yearly out of pocket costs increased by $1000 to $5,000
05 My yearly premium and/or yearly out of pocket costs increased by more than $5,000
06 Do not recall

ASK IF Q44A1=01

Q44a4. Since you changed from full-time to part-time employment, have you made any **significant changes in your living arrangement or life style**? (*Mark all that apply*)

01 Made modification in living arrangements (e.g., sold house, downsized house, or rental)
02 Cut back on everyday living costs (e.g., clothes, food, car)
03 Filed for bankruptcy
04 Other (Please specify)

ASK IF Q40=01

Q45. About **the most recent time you changed from full-time to part-time employment** due to your [**S2A**]:

Did you ever return to full-time, whether at that job or another job?

01 Yes
02 No

ASK IF Q45=01

Q46. About **the most recent time you changed from full-time to part-time employment** due to your [**S2A**]:

How long did it take you to return to full-time, whether at that job or another job?

Years (NOT REQUIRED) allow blanks without validation)	Months (NOT REQUIRED – allow blanks without validation)
//RANGE=1-70//	//RANGE=0-11//

ASK IF Q5= 01 OR 02

Q47. As a result of your [S2A], have you ever had **any other reductions in your hours at work for 3 months or more**?

01 Yes
02 No

ASK IF Q47=01

Q48. How many times have you ever had **any other reductions in your hours at work for 3 months or more** as a result of your [S2A]?

01 1 time
02 2 times
03 More than 2 times

ASK IF Q48=01

Q49a.

Please enter the year that you had **other reductions in your hours at work for 3 months or more** as a result of your [S2A].	//RANGE=1945-2016//

ASK IF Q48=02

Q49b.

Please enter the year for the 1st time you **had other reductions in your hours at work for 3 months or more** as a result of your [S2A].	//RANGE=1945-2016//
Please enter the year for the 2nd time you **had other reductions in your hours at work for 3 months or more** as a result of your [S2A].	//RANGE=1945-2016//

ASK IF Q48=03

Q49c. Please enter the years for the times you **had other reductions in your hours at work for 3 months or more** as a result of your {{ Q3 }}.

What was the year for the 1st time?	//RANGE=1945-2016//
What was the year for the 2nd time?	//RANGE=1945-2016//
What was the year for the most recent time?	//RANGE=1945-2016//

The following questions ask about **the most recent time when you reduced work hours** due to your **[S2A].**

ASK IF Q47=01

Q50. How much was your annual salary/wage at the time you reduced your hours?

[same format for other income/salary questions]

ASK IF Q47=01

Q51. About the **most recent time when you reduced work hours** due to your [S2A]:
How many hours a week (on average) did you work before your hours were reduced ?
[RANGE=1-60]____________

ASK IF Q47=01

Q52. About the **most recent time when you reduced work hours** due to your [S2A]:
How many hours a week (on average) did you work after your hours were reduced ?
[RANGE=1-60] ____________

ASK IF Q47=01

Q53. About the **most recent time when you reduced work hours** due to your [S2A]:

How much was your annual salary/wage after your hours were reduced?

[same format for other income/salary questions]

ASK IF Q47=01

Q53a1. Did you experience a **change in health insurance coverage** when you reduced your work hours?
01 Yes
02 No

ASK IF Q53A1=01

Q53a2. What was the **change in health insurance coverage**?
01 Switched from employer sponsored plan to COBRA
02 Switched from employer sponsored plan to Medicare or Medicaid
03 Switched from employer sponsored plan to self-purchased insurance
04 Switched to a new employer sponsored insurance plan
05 Became uninsured
06 Other

ASK IF Q53A1=01

Q53a3. Compared to your previous health insurance coverage, did your **new insurance costs change**?
01 Paid less for my yearly premium and out of pocket costs (e.g., copay, deductible)
02 No change in insurance premium and out of pocket costs
03 My yearly premium and/or yearly out of pocket costs increased by $1 to $1000
04 My yearly premium and/or yearly out of pocket costs increased by $1000 to $5,000
05 My yearly premium and/or yearly out of pocket costs increased by more than $5,000
06 Do not recall

ASK IF Q53A1=01

Q53a4. Since you reduced your work hours, have you made any **significant changes in your living arrangement or life style**? (*Mark all that apply*)

01 Made modification in living arrangements (e.g., sold house, downsized house, or rental)
02 Cut back on everyday living costs (e.g., clothes, food, car)
03 Filed for bankruptcy
04 Other (Please specify)

ASK IF Q47=01

Q54. About the **most recent time when you reduced work hours** due to your [S2A]:
Did you ever return to your previous work week hours, either at that job or another job?

01 Yes
02 No

ASK IF Q54=01

Q55. About the **most recent time when you reduced work hours** due to your [S2A]:

How long did it take you to return to your previous work week hours, either at that job or another job?

Years (NOT REQUIRED) allow blanks without validation)	Months (NOT REQUIRED – allow blanks without validation)
//RANGE=1-70//	//RANGE=0-11//

ASK IF Q5= 01 OR 02

Q56. As a result of your [S2A], were you ever **reassigned to or did you take another job at a lower salary or wage**?

01 Yes
02 No

ASK IF Q56=01

Q57. How many times have you ever been **reassigned to or did you take another job at a lower salary or wage** as a result of your [S2A]?

01 1 time
02 2 times
03 More than 2 times

ASK IF Q56=01

Q58a.

Please enter the year that you were **reassigned to or took another job at a lower salary or wage** as a result of your [S2A].	//RANGE=1945-2016//

ASK IF Q56=02

Q58b.

Please enter the year for the 1st time you were **reassigned to or took another job at a lower salary or wage** as a result of your [S2A].	//RANGE=1945-2016//
Please enter the year for the 2nd time you were **reassigned to or took another job at a lower salary or wage** as a result of your [S2A].	//RANGE=1945-2016//

ASK IF Q56=03

Q58c. Please enter the years for the times you **had other reductions in your hours at work for 3 months or more** as a result of your [S2A].

What was the year for the 1st time?	//RANGE=1945-2016//
What was the year for the 2nd time?	//RANGE=1945-2016//
What was the year for the most recent time?	//RANGE=1945-2016//

The following questions ask about **the most recent time you were reassigned to another job by an employer at a lower salary/wage** due to your [S2A].

ASK IF Q56=01

Q59. Was your job prior to reassignment full-time or part-time?

01 Full-time
02 Part-time

ASK IF Q56=01

Q60. About **the most recent time you were reassigned to another job by an employer at a lower salary/wage** due to your [S2A]:

How much was your annual salary/wage at the time you were reassigned to or took another lower paying job?

[same format for other income/salary questions]

ASK IF Q56=01

Q61. About **the most recent time you were reassigned to another job by an employer at a lower salary/wage** due to your **[**S2A**]:**

Was the lower paying job a full-time or part-time job?

01 Full-time

02 Part-time

ASK IF Q56=01

Q62. About **the most recent time you were reassigned to another job by an employer at a lower salary/wage** due to your **[**S2A**]:**

Was the lower paying job a less demanding one?

01 Yes

02 No

ASK IF Q56=01

Q63. About **the most recent time you were reassigned to another job by an employer at a lower salary/wage** due to your **[**S2A**]:**

How much was your annual salary/wage at the new job?

[same format for other income/salary questions]

ASK IF Q56=01

Q64. About **the most recent time you were reassigned to another job by an employer at a lower salary/wage** due to your **[**S2A**]:**

Did you ever return to your previous annual salary, either at that job or another job?

01 Yes

02 No

ASK IF Q64=01

Q65. About the **most recent time when you reduced work hours** due to your [**S2A**]:

How long did it take for you to return to your previous annual salary, either at that job or another job?

Years (NOT REQUIRED) allow blanks without validation)	Months (NOT REQUIRED – allow blanks without validation)
//RANGE=1-70//	//RANGE=0-11//

ASK ALL

Q66. Which of the following treatments have you ever received for your [S2A]? (*Mark all that apply*)

Treatment	Check Box
Q66_1. Bone marrow transplant or stem cell transplant	
Q66_2. Phlebotomy	
Q66_3. Removal of spleen	
Q66_4. Blood transfusion	
Q66_5. Droxia, Hydrea (hydroxyurea, hydroxycarbamide)	
Q66_6. Jakafi® (ruxolitinib)	
Q66_7. Pegasys, Peg-Intron (interferon)	
Q66_8. Agrylin (anagrelide)	
Q66_9. Aspirin	
Q66_10. Anticoagulants or blood thinner (for example, warfarin, Coumadin)	
Q66_11. Radiation	
Q66_12. No treatment	
Q66_13. Other Investigational agent (please specify)	

ASK IF Q66_1, Q66_2, Q66_3, Q66_4, Q66_5, Q66_6, Q66_7, Q66_8, Q66_9, Q66_10, Q66_11, Q66_13=01

Q67. Which of the following is/are your most recent treatment(s)? (*Mark all that apply*)
[SHOW ONLY OPTIONS SELECTED IN Q66]

Treatment	Radio button selection =01(yes)
[ASK IF Q66_1=01] Q67_1. Bone marrow transplant or stem cell transplant	
[ASK IF Q66_2=01] Q67_2. Phlebotomy	
[ASK IF Q66_3=01] Q67_3. Removal of spleen	
[ASK IF Q66_4=01] Q67_4. Blood transfusion	
[ASK IF Q66_5=01] Q67_5. Droxia, Hydrea (hydroxyurea, hydroxycarbamide)	
[ASK IF Q66_6=01] Q67_6. Jakafi (ruxolitinib)	
[ASK IF Q66_7=01] Q67_7. Pegasys, Peg-Intron (interferon)	
[ASK IF Q66_8=01] Q67_8. Agrylin (anagrelide)	
[ASK IF Q66_9=01] Q67_9. Asprin	
[ASK IF Q66_10=01] Q67_10. Anticoagulants or blood thinner (for example,warfarin, Coumadin, etc)	
[ASK IF Q66_11=01] Q67_11. Radiation	
[ASK IF Q66_13=01]. Q67_13. Other Investigational agent	

ASK IF Q66_1, Q66_2, Q66_3, Q66_4, Q66_5, Q66_6, Q66_7, Q66_8, Q66_9, Q66_10, Q66_11, Q66_13=01

Q68. When did you start your most recent or last treatment?

01 Less than one month ago
02 1 to 3 months ago
03 3 to 6 months ago
04 6 to12 months ago
05 More than 12 months ago

ASK IF Q66_1, Q66_2, Q66_3, Q66_4, Q66_5, Q66_6, Q66_7, Q66_8, Q66_9, Q66_10, Q66_11, Q66_13=01

Q69. What was your employment situation when you started your most recent treatment for [**S2A**]? [**REQUIRED**]

01 Employed full-time (40 hours a week)
02 Employed part-time (less than 40 hours a week)
03 Unemployed seeking employment
04 Unemployed not seeking employment
05 Retired
06 Self-employed
07 Disabled
09 Home maker
08 Other

ASK ALL

Q70. What is your current employment situation? **[REQUIRED]**

01 Employed full-time (40 hours a week)
02 Employed part-time (less than 40 hours a week)
03 Unemployed seeking employment
04 Unemployed not seeking employment
05 Retired
06 Self-employed
07 Disabled
09 Home maker
08 Other

Q71. In your opinion, what do you think your current employment situation would have been if you had started this **most recent treatment** earlier?

My current employment situation would likely have been:
[REMOVE ANSWER TO Q70 FROM RESPONSE LIST]

10 The same as it is now
01 Employed full-time (40 hours a week)
02 Employed part-time (less than 40 hours a week)
03 Unemployed, seeking employment
04 Unemployed, not seeking employment
05 Retired
06 Self-employed
07 Disabled
08 Home maker
09 Retired, but would not have taken early retirement

ASK IF [Q5=01 OR 02 OR 06]

Q72. Since your most recent treatment, how much has your [S2A] impacted your productivity at work?

01 My productivity has improved a lot
02 My productivity has improved a little
03 There has been no change to my productivity
04 My productivity has decreased a little
05 My productivity has decreased a lot

ASK IF Q70= 01, 02 OR 06

Q73. Which best describes your current profession?

01 Executive or manager
02 Professional
03 Technical support
04 Sales
05 Clerical and administrative support
06 Service occupation
07 Skills/Crafts worker
08 Laborer
09 Other

ASK IF Q70= 01,02 OR 06

Q74. Which best describes the industry in which you work?

01 Accommodation and food services
02 Agriculture, forestry, and fishing
03 Arts and entertainment
04 Construction
05 Education
06 Finance
07 Health and social care
08 Information
09 Manufacturing
10 Professional, scientific, and technical services
11 Public services
12 Retail/Trade
13 Transportation
14 Utilities
15 Other

ASK ALL

Q75. As a result of your [**S2A**],

[REQUIRED]

	Not at all	A little	Somewhat	Quite a bit	A great deal
have you ever been limited in your career opportunities?					
have you ever been limited in your wages/salary (from employment, investment, etc.)?					
have you ever been limited in your ability to pursue certain types of jobs or careers					
have you ever been forced to change your career choices?					

Q76. Since your diagnosis, to what extent did your [S2A] impact the amount of time you spent [REQUIRED]

	Not at all	A little	Somewhat	Quite a bit	A great deal
with friends and family?					
doing leisure activities (e.g., reading, watching television, sports)?					
doing work around the house (e.g., cleaning, yardwork)?					

ASK ALL

Q77. How often do you rely on someone (i.e., caregiver) to assist you with your activities of daily living due to your [S2A] condition:

Assistance with activities of daily living can range from a few hours of shopping and cleaning to intensive medical or personal care. Tasks can include shopping, house cleaning, cooking, giving medications, toileting assistance and so forth. The person who assists you with your activities of daily living will be referred to as "**caregiver**" in the following survey.

01 Never
02 Rarely
03 Sometimes
04 Often

ASK IF Q77=02,03,04

Q78. Who is the main caregiver who helps you due to your [S2A]?

01 Spouse/partner
02 Parent
03 Son/Daughter
04 Sibling
05 Other relative
06 Paid nurse or home healthcare professional
07 Other

ASK IF Q77=02,03,04

Q79. What kind of help do you require from your main caregiver due to your [S2A]?
Mark all that apply
01 Companionship (e.g., talking, reading, keeping company) or supervision
02 Transportation (e.g., driving to doctor's appointments, driving for errands)
03 Homemaking (e.g., shopping, cleaning, preparing meals)
04 Personal care assistance (e.g., feeding, bathing, toileting, dressing, grooming)
05 Healthcare assistance (e.g., help with medications, wound care)
06 Managing finances (e.g., paying bills, managing budget)

ASK IF Q77=02,03,04

Q80. In the past 7 days, how many total hours has your main caregiver spent helping you with these activities because of your [S2A]?

________ [Enter number of hours] //RANGE=0-720//

ASK IF Q78=06

Q81. In the past 7 days, how much have you spent out-of-pocket on the nurse or home healthcare professional to help you with your [S2A]?

$ __________ [Enter numerical value] //RANGE=0-1,000,000//

ASK IF Q78=01,02,03,04,05, 07

Q82. Was your main caregiver employed at the time of your diagnosis?

01 Yes
02 No

Q78=01,02,03,04,05, 07

Q83. As a result of your [S2A] has your caregiver ever reduced his/her hours at work? [REQUIRED]
01 Yes
02 No
03 Not applicable

Q84. As a result of your [S2A] has your caregiver ever terminated his/her job? [REQUIRED]
01 Yes
02 No
03 Not applicable

Q85. As a result of your [S2A] has your caregiver ever taken early retirement? [REQUIRED]
01 Yes
02 No
03 Not applicable

Q86. As a result of your [S2A] has your caregiver ever considered terminating his/her job (but did not terminate for reasons such as health insurance coverage, etc.)? **[REQUIRED]**

01 Yes
02 No
03 Not applicable

ASK ALL

Q87. What was your annual income last year (before taxes; please include wage/salary and other income; not including income from other family members)?

[same format for other income/salary questions]

ASK ALL

Q88. Have there been any major decreases to your income as a result of your [S2A] over the past 12 months?

01 Yes
02 No

ASK IF Q88=01

Q89. Please specify the decrease to your income (before taxes) as a result of your [S2A] over the past 12 months.

[same format for other income/salary questions]

ASK ALL

Q89a1. Have you had to pay additional costs for any of the following related to your [S2A]? (*Mark all that apply*)

01 Medical devices (such as a wheelchair)
02 Nutritional supplements
03 Structural changes to housing (e.g., bathroom, stair lift)
04 Help (e.g., baby sitter, cleaning person)
05 I paid no additional costs
06 Other [SPECIFY]

ASK IF ANY SELECTED IN Q89A1

Q89a2. Please enter the amount, as best you can, that you have paid out of pocket as a result of your [S2A] in the past 12 months.

[WRITE-IN TEXT BOX]

ASK ALL

Q90. Over the past 7 days, how severe was the [symptom] associated with your [S2A] on a scale from 0 (absent) to 10 (worst imaginable). **[REQUIRED] [EACH ITEM WILL BE DISPLAYED ON ITS OWN PAGE WITH THE FOLLOWING RESPONSE SCALE]**

[IF RESPONSE SCALE IS HORIZONTAL, DISPLAY FOLLOWING MESSAGE] If you are taking this survey on an iPad or smart phone please hold your device horizontally to complete the items.

	**Absent**										**Worst Imaginable**
	0	1	2	3	4	5	6	7	8	9	10
Abdominal discomfort (feeling uncomfortable, pressure, or bloating)											
Abdominal pain (under the ribs on the left side)											
Bone pain (diffuse, not joint pain or arthritis)											
Bruising											
Cough											
Day or night sweats											
Dizziness/vertigo/lightheadedness											
Problems with vision (e.g., double vision, blurred vision)											
Facial flushing											
Fatigue or tiredness											
Fever (>100°F)											
Filling up quickly when you eat (early satiety)											
Itching											
Muscle aches											
Night sweats											
Nosebleeds											
Numbness/Tingling in your hands and feet											
Headaches											
Shortness of breath											
Unintentional weight loss											
Problems with concentration											
Inactivity											

ASK ALL

Q91. Over the past 7 days, to what extent did your [S2A] cause you: **[REQUIRED]**
[EACH ITEM WILL BE DISPLAYED ON ITS OWN PAGE WITH THE FOLLOWING RESPONSE SCALE]

	Not at all	A little	Somewhat	Quite a bit	A great deal
To have difficulty sleeping					
To need to rest more often					
To feel depressed or sad					
To feel anxious or worried about your future					
To feel irritable					
To be limited in your ability to socialize (meal with friends, etc.)					
To feel isolated					
To have problems with concentrating on usual tasks					
Difficulty remembering things					
Difficulty with strenuous physical activities (lifting things, long walks, etc.)					
Difficulty with your daily activities (e.g., running errands)					
Difficulty with self-care (e.g., dressing, washing, using the toilet)					
Difficulty doing work around the house (e.g., cleaning, yardwork, etc.)					
Difficulty in attending major social events (graduations, weddings, etc.)					
Problems with your sex life					

The following questions ask about the effect of your [S2A] on your ability to work and perform regular activities. The data collected in this section can be used to describe [S2A] disease impact on work/productivity as compared with other diseases.

Please fill in the blanks or choose a number, as indicated.

The questions are about the **past 7 days**, not including today.

ASK IF Q70 = 01 OR 02 (IF NOT -> SKIP TO Q95)

Q92. During the past 7 days, how many hours did you miss from work because of problems associated with your[S2A]? *Include hours you missed on sick days, medical appointment, times you went in late, left early, etc., because of your* [S2A]*]. Do not include time you missed to participate in this study.*

_____ HOURS//RANGE=0-168//

ASK IF Q70 = 01 OR 02

Q93. During the past 7 days, how many hours did you actually work?

_____HOURS //RANGE=0-168//

ASK IF Q70 = 01 OR 02

Q94. During the past 7days, how much did your [S2A] affect your productivity while you were working?

Think about days you were limited in the amount or kind of work you could do, days you accomplished less than you would like, or days you could not do your work as carefully as usual. If [S2A]? affected your work only a little, choose a low number. Choose a high number if [S2A] affected your work a great deal.

Consider only how much [S2A] affected productivity while you were working.

[S2A] had no effect on my work	0	1	2	3	4	5	6	7	8	9	10	[S2A]completely prevented me from working

ASK ALL

Q95. During the past 7 days, how much did your [S2A] affect your ability to do your regular daily activities, other than work at a job?

By regular activities, we mean the usual activities you do(work around the house, shopping, childcare, exercising, studying, etc.). Think about times you were limited in the amount or kind of activities you could do and times you accomplished less than you would like. If [S2A] affected your activities only a little, choose a low number. Choose a high number if [S2A] affected your activities a great deal.

Consider only how much [S2A] affected your ability to do your regular daily activities, other than work at a job.

[S2A] had no effect on my daily activities	0	1	2	3	4	5	6	7	8	9	10	[S2A] completely prevented me from doing my daily activities

ASK ALL

PRE6. The following questions ask about your demographic profile, which are not disease related.

Q96. What is your marital status?
01 Single, never married
02 Married or domestic partnership
03 Widowed
04 Divorced
05 Separated

ASK ALL

Q97. What is your current household size?
___________ number of people who live in the household [RANGE 1-50]

ASK ALL

Q98. Do you have any sort of health insurance or health plan to cover your health care costs?
01 Yes
02 No

ASK IF Q24=01

Q99. What best describes your type of primary coverage?

01 Group commercial insurance through employer or union
02 Individual commercial insurance that is self-paid
03 Medicare
04 Medicaid or State assistance
05 Tricare or VA benefit
06 Other

ASK ALL

Q100. Are you currently participating in a clinical trial for [S2A]? [REQUIRED]

1. Yes
2. No

Completion Page

Thank you for your participation. Please complete the following information to receive your $25 Visa gift card sent to you by mail :

Note: Your contact information will ONLY be used for the purposes of this study.

◯ I choose not to receive gift card for my participation

◯ I choose to receive gift card for my participation

ASK IF THEY CHOOSE TO RECEIVE PAYMENT [REQUIRED if choosing payment]

Insert Name ______________________________

Address ______________________________

City ______________________________

State ______________________________

Zip Code ______________________________

E-mail Address ______________________________

TERM FINAL You have completed the survey. Thank you so much for your time. You may close this browser window at any time.
